# Deficiency of pigment epithelium-derived factor in nasopharyngeal carcinoma cells triggers the epithelial–mesenchymal transition and metastasis

**DOI:** 10.1038/cddis.2017.114

**Published:** 2017-06-01

**Authors:** Ting Zhang, Ping Yin, Zichen Zhang, Banglao Xu, Di Che, Zhiyu Dai, Chang Dong, Ping Jiang, Honghai Hong, Zhonghan Yang, Ti Zhou, Jianyong Shao, Zumin Xu, Xia Yang, Guoquan Gao

**Affiliations:** 1 Program of Molecular Medicine, Affiliated Guangzhou Women and Children’s Hospital, Zhongshan School of Medicine, Sun Yat-sen University, Guangzhou 510080, China; 2 Department of Biochemistry, Zhongshan School of Medicine, Sun Yat-sen University, Guangzhou 510080, China; 3 Department of Laboratory Medicine, Guangzhou First People’s Hospital, Guangzhou Medical University, Guangzhou 510180, China; 4 Department of Molecular Diagnostics, Sun Yat-sen University Cancer Center, Guangzhou 510160, China; 5 Department of Clinical Laboratory, Third Affiliated Hospital of Guangzhou Medical University, Guangzhou 510150, China; 6 Cancer Center, Affiliated Hospital of Guangdong Medical College, Zhanjiang 524001, China; 7 Guangdong Engineering & Technology Research Center for Gene Manipulation and Biomacromolecular Products (Sun Yat-sen University), Guangzhou 510080, China; 8 China Key Laboratory of Tropical Disease Control (Sun Yat-sen University), Ministry of Education, Guangzhou 510080, China

## Abstract

Distant metastasis is the primary cause of nasopharyngeal carcinoma (NPC) treatment failure while epithelial–mesenchymal transition (EMT) is the critical process of NPC invasion and metastasis. However, tumor-suppressor genes involved in the EMT and metastasis of NPC have not been explored clearly compared with the oncogenes. In the present study, the expression of pigment epithelium-derived factor (PEDF), a potent endogenous antitumor factor, was diminished in human NPC tissues and associated with clinicopathological and EMT features. The knockdown of PEDF induced EMT in lower metastatic NPC cell lines and overexpression of PEDF restored epithelial phenotype in higher metastatic NPC cell lines with typical EMT. The inhibition of PEDF mediated NPC cell spontaneous metastasis *in vivo*. LRP6/GSK3*β*/*β*-catenin signal pathway rather than AKT/GSK3*β* pathway was involved in the effects of PEDF on EMT. The expression of PEDF was directly downregulated by elevated miR-320c in NPC. In conclusion, our findings indicate for the first time that PEDF functions as tumor-suppressor gene in the occurrence of EMT and metastasis in NPC. PEDF could serve as a promising candidate for NPC diagnosis, prognosis and treatment.

Nasopharyngeal carcinoma (NPC) is prevalent in southern China and South-East Asia, with an annual incidence rate of about 25–30 per 100 000 people, wheras it is rare in the Western world (1 per 100 000).^1^ Although NPC is sensitive to radiotherapy and chemotherapy, the overall 5-year survival rate of NPC patients is around 80%, 20–30% patients develop distant metastasis or loco-regional recurrence eventually leading to death.^2^ Therefore, understanding the mechanism of NPC metastasis and identification of effective anti-nasopharyngeal carcinoma metastasis drugs have been emerging as a promising direction for the treatment of NPC.

Recent studies have shown that epithelial–mesenchymal transition (EMT) is closely related to tumor metastasis. EMT occurs in embryogenesis, fibrosis and invasion of the tumor. They have many common characteristics, such as the loss of the connection between epithelial cells and epithelial cell phenotypic markers, increases mesenchymal markers and endows cell migration ability.^3^ EMT is closely related to the invasion and metastasis of NPC as well, inhibition of NPC cell’s EMT could significantly suppress the metastasis of NPC.^4, 5, 6, 7^

The canonical Wnt/*β*-catenin pathway is involved in various biological processes, including embryonic development, stem cell maintenance and tumorigenesis. When Wnt/*β*-catenin pathway was activated, the core protein *β*-catenin translocates into cell nucleus and directly involve in gene transcription and cell adhesion.^8^ Zeng *et al.*^9^ and co-workers observed Wnt pathway was abnormally activated in NPC using nasopharyngeal tissue array. It has been reported that activation of Wnt/*β*-catenin pathway by oncogenes could promote EMT and metastasis in NPC.^10, 11, 12^ However, tumor-suppressor genes involved in the EMT and metastasis of NPC have not been identified.

Pigment epithelium-derived factor (PEDF) is a potent and versatile endogenous inhibitor of angiogenesis.^13^ Previous studies demonstrated that PEDF is a favorable prognostic indicator in colorectal, pancreatic, lung and breast cancer.^14, 15, 16, 17^ There is a complex mechanism underlying the antitumor effects of PEDF, which includes inhibition of angiogenesis and tumor cell migration, induction of apoptosis and pro-tumor differentiation in certain tumor cell types.^18^ However, it is still unclear the exact role of endogenous PEDF in EMT occurrence and NPC metastasis. Previously, we have reported that PEDF could bind to low-density lipoprotein receptor-related protein 6 (LRP6) and hinder the Wnt ligand-induced dimerization of LRP6 and Fz receptor, thus block the activation of canonical Wnt signaling in diabetic complications.^19, 20^ It is unknown whether PEDF could also block Wnt/*β*-catenin activation and further suppress EMT and metastasis in NPC.

From the clinical tissue chips analysis, we found the extremely low expression of PEDF in NPC was closely associated with advanced clinicopathological stage. Therefore, the hypothesis of PEDF as a possible tumor-suppressor gene and the critical role of PEDF deficiency in the occurrence of EMT and metastasis in nasopharyngeal carcinoma cells will be explored. Further, the underlying mechanism of PEDF on EMT and the expression regulation of PEDF will also be investigated in the present study.

## Results

### PEDF expression is diminished in human NPC cells, associated with clinicopathological and EMT features

**Figure 1:**
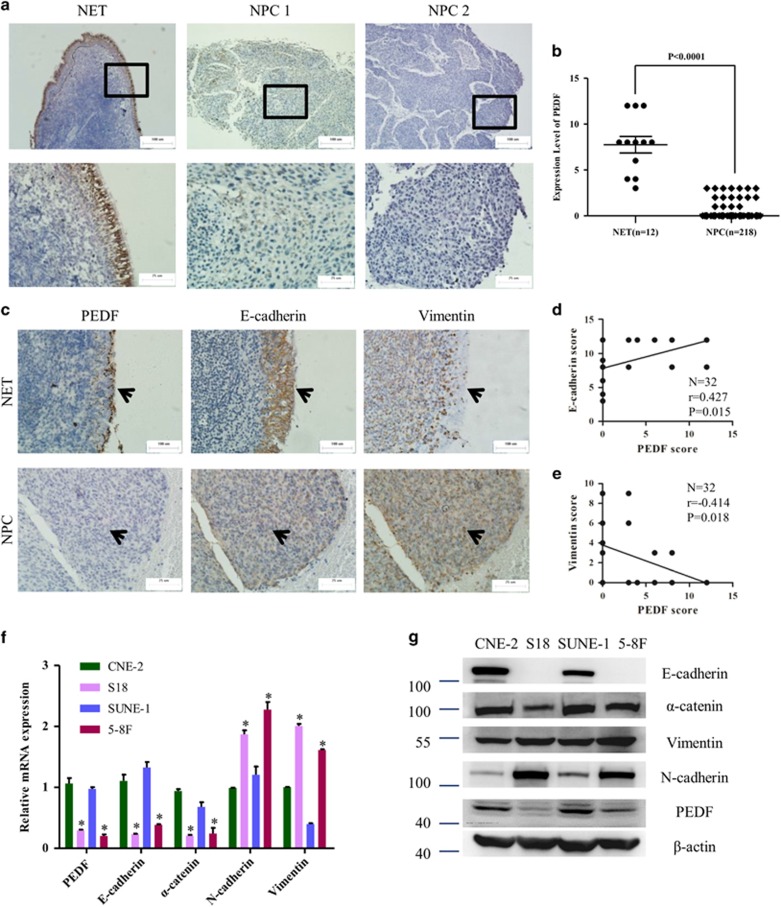
Pigment epithelium-derived factor (PEDF) expression is diminished in human nasopharyngeal carcinoma (NPC) cells, associated with clinicopathological and epithelial–mesenchymal transition (EMT) features. (**a**) The representative area of nasopharynx epithelial tissues (NET) or NPC. NPC 1, weak PEDF expression. NPC 2, negative PEDF expression. (**b**) Quantitative analysis of staining intensity for PEDF in various tissues. (**c**) The expression of PEDF, E-cadherin and Vimentin in a cohort of NET biopsy samples and NPC samples using immunohistochemistry assay. (**d** and **e**) PEDF significantly correlates with E-cadherin and Vimentin in a cohort of NET biopsy samples and NPC samples. (**f** and **g**) Real-time PCR and western blot (WB) analysis of the expression levels of PEDF in a series of human NPC cell lines, normalized to *β*-actin levels. Bars correspond to mean±standard deviation (S.D.), *P<0.05

To determine whether PEDF expression was modulated during nasopharyngeal carcinoma progression, we measured PEDF expression in the nasopharynx epithelial tissues (NET) and NPC tissues (Figure 1a). The clinical characteristics of 218 NPC patients were presented in Supplementary Table 3. All (12/12) of NET showed strong positive staining, but only 7.3% (16/218) of NPC tumor tissues had weak staining (Figure 1b). Next, the tumors were categorized as positive or negative for PEDF. We observed NPC patients of negatively PEDF expression had advanced pathological tumor stage and clinical stage. No significant correlation between PEDF expression and pathological node stage was observed (Table 1). EMT is the critical process of NPC invasion and metastasis. To explore the relationship between PEDF and EMT, we performed immunohistochemical staining of PEDF, E-cadherin and vimentin in another 12 NET samples and 20 NPC biopsies. As shown in Figures 1c–e, PEDF expression level positively correlated with the expression level of E-cadherin (*r*=0.427, *P*<0.05), and inversely related to the expression level of vimentin (*r*=−0.414, *P*<0.05). Next, real-time PCR and western blot was performed to quantify the levels of PEDF among a series of NPC cells. Results showed low-metastatic CNE-2 and SUNE-1 cells had high PEDF expression, which was accompanied by reduced levels of mesenchymal markers vimentin and N-cadherin and elevated levels of epithelial proteins E-cadherin and *α*-catenin compared with high-metastatic S18 and 5–8F cells (Figures 1f and g). All these data suggested that PEDF might have an inhibitory effect on EMT.

### Knockdown of endogenous PEDF expression in NPC cells induces EMT

**Figure 2:**
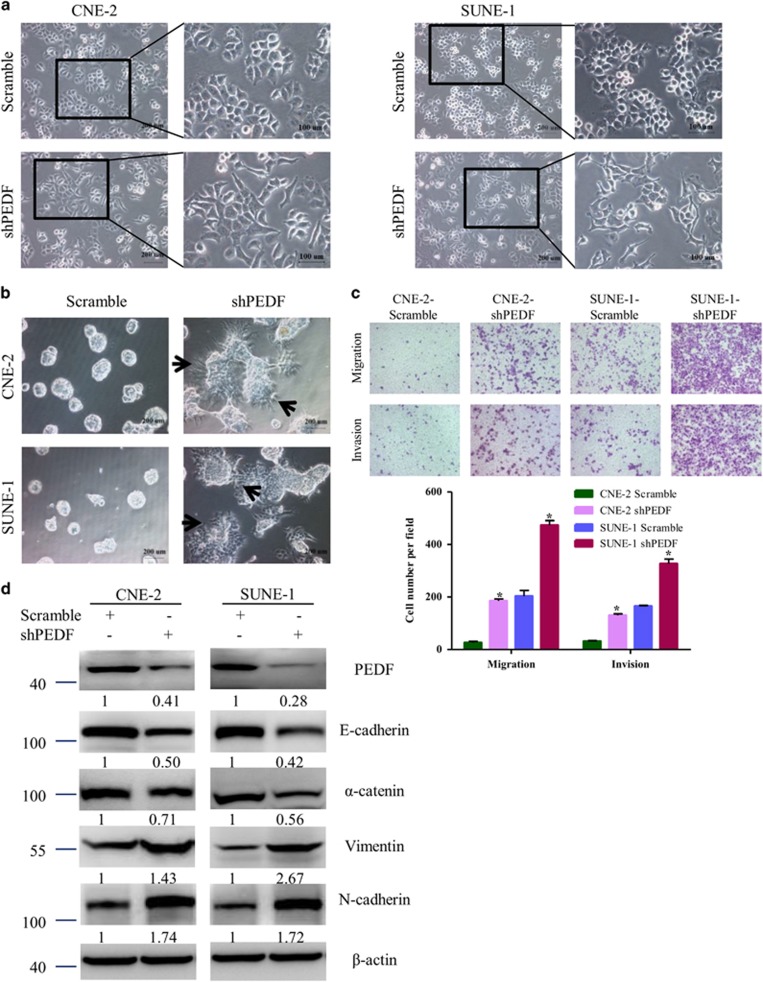
Knockdown of endogenous PEDF expression in NPC cells induces EMT. (**a**) Cell morphology was evaluated by phase-contrast microscopy. (**b**) Representative micrographs of indicated cells grown on Matrigel for 8 days in 3D spheroid invasion assay. (**c**) The migration/invasion ability of indicated cells was evaluated by Transwell assay. (**d**) Expression of epithelial cell markers (E-cadherin, *α*-catenin) and mesenchymal cell markers (Vimentin, N-cadherin) in indicated cells were examined by WB. *β*-actin was used as a loading control. Bars correspond to mean±standard deviation (S.D.), *P<0.05

To examine the causal role of PEDF in EMT of NPC cells, we engineered cell lines from low-metastatic CNE-2 and SUNE-1 cells that stably expressed either shRNA targeting PEDF expression (shPEDF) or a scrambled nontarget shRNA. As shown in Figure 2a, knockdown of PEDF led to the transformation of the cobblestone-like cells CNE-2 and SUNE-1 to spindle-like, fibroblastic cells. Furthermore, in 3D culture, PEDF-knockdowned CNE-2 and SUNE-1 cells grew into more structurally well-organized spheres with invasive projections emanating from the cells when compared with the scrambled control cells (Figure 2b). We also observed that the invasive and migratory properties were enhanced upon silencing PEDF (Figure 2c). Also, the typical EMT markers were changed includes the downregulation of epithelial markers E-cadherin and *α*-catenin, upregulation of mesenchymal markers vimentin and N-cadherin (Figure 2d). These results proved that knockdown of PEDF is sufficient to induce EMT in NPC cells.

### Overexpression of PEDF induces restoration of the epithelial phenotype

**Figure 3:**
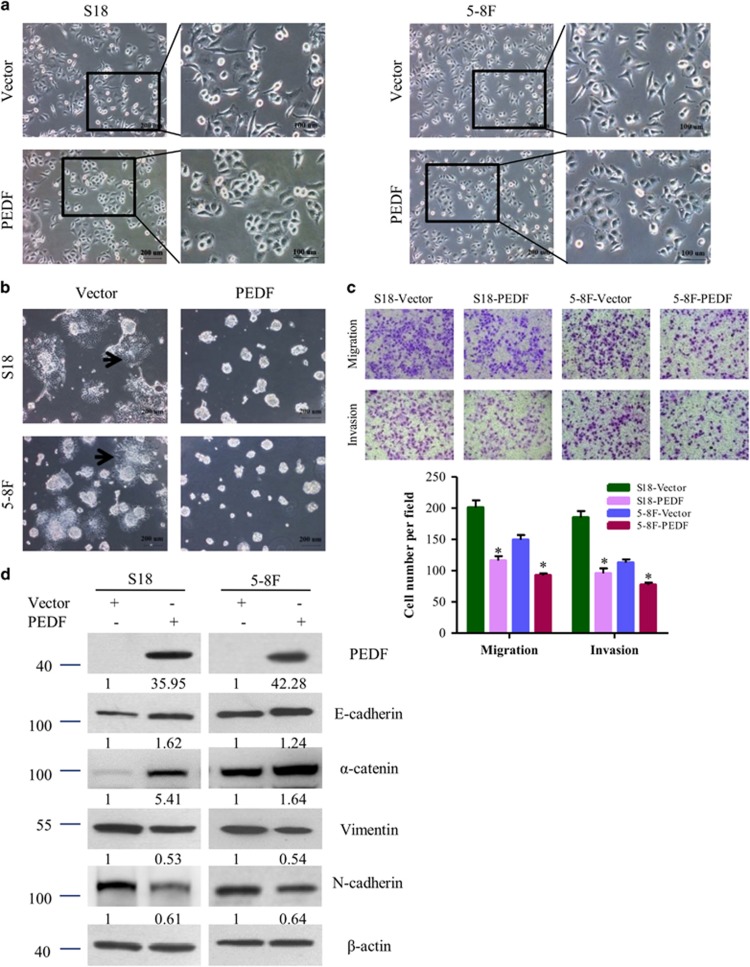
Overexpression of PEDF induces restoration of the epithelial phenotype. (**a**) Cell morphology was evaluated by phase-contrast microscopy. (**b**) Representative micrographs of indicated cells grown on Matrigel for 8 days in 3D spheroid invasion assay. (**c**) The migration/invasion ability of indicated cells was evaluated by Transwell assay. (**d**) Expression of epithelial cell markers (E-cadherin, *α*-catenin) and mesenchymal cell markers (Vimentin, N-cadherin) in indicated cells were examined by WB. *β*-Actin was used as a loading control. Bars correspond to mean±standard deviation (S.D.), *P<0.05

To further investigate the impact of PEDF on EMT in NPC cells, we overexpress PEDF in the high-metastatic S18 and 5–8 F cells. As shown in Figure 3a, S18 and 5–8F cells were transformed from spindle-lie and fibroblastic phenotype to cobblestone-like after overexpressing PEDF. In 3D culture, S18/Vector and 5–8 F/Vector cells grew into more structurally well-organized spheres with invasive projections emanating from the cells when compared with the S18/PEDF and 5–8 F/PEDF cells (Figure 3b). The migration and invasion abilities of S18 and 5–8 F cells were also reduced in PEDF-overexpressing cells (Figure 3c). Western blot confirmed that reduced expression of the epithelial markers, increased expression of the mesenchymal markers upon PEDF overexpression (Figure 3d). These results confirmed that PEDF could inhibit NPC cells migration and invasion through suppression of EMT.

### Knockdown of PEDF mediates the distant metastasis *in vivo*

**Figure 4:**
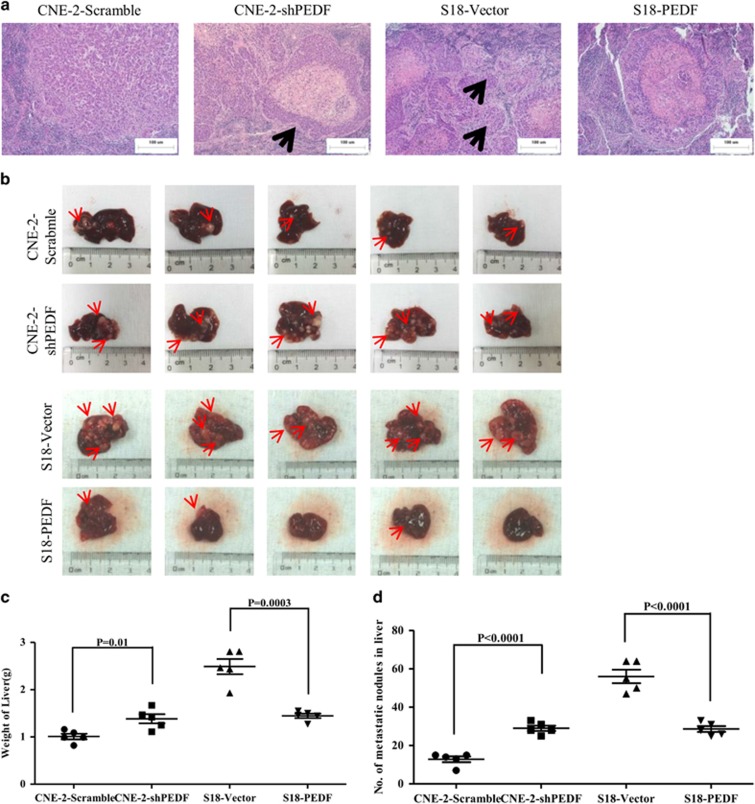
PEDF inhibits the spontaneous liver metastasis of NPC cells *in vivo*. (**a**) Representative H&E staining of indicated spleen orthotopic tumors was shown. Arrows indicate the small metastases. (**b**) Representative bright-field image of the livers was shown. Arrows indicate surface metastatic nodules. (**c** and **d**) Liver weights and metastatic modules number in nude mice that received transplants of indicated cells. Bars correspond to mean±standard deviation (S.D.), *P<0.05

As the low PEDF expression was associated with NPC metastatic traits, we next asked whether PEDF could mediate NPC cells metastasis *in vivo*. The effect of PEDF *in vivo* was determined by using a spontaneous spleen transfer to liver model. We found mice bearing CNE-2/shPEDF and S18/Vector primary tumors displayed more invasive phenotype, which included the NPC cells invaded into the normal spleen tissues and formed small metastasis, compared with nude mice injected with CNE-2/scramble or S18/PEDF cells (Figure 4a). As expected, PEDF knockdown cells showed a significant increased liver metastasis and weight, and the liver metastasis was dramatically inhibited as well as the weight of liver was decreased by overexpressing PEDF (Figures 4b–d). All of these data confirmed that PEDF has a pivotal role in NPC metastasis *in vivo*.

### Wnt/*β*-catenin signaling pathway is required for PEDF-mediated EMT

**Figure 5:**
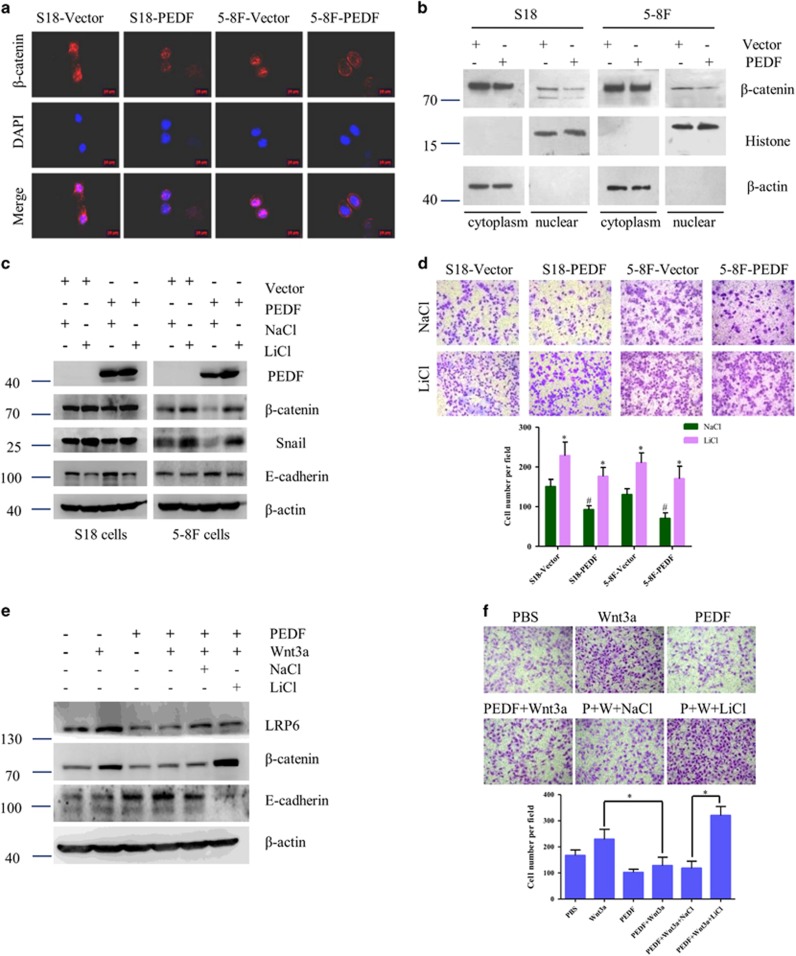
Wnt/*β*-catenin signaling pathway is required for PEDF-mediated EMT. (**a**) Subcellular *β*-catenin localization in indicated cells was assessed by immunofluorescence staining. (**b**) Nuclear fractions of indicated cells were analyzed by WB analysis. (**c** and **d**) Cells were treated with Wnt/*β*-catenin signaling agonist LiCl (50 nM) for 16 h, and then performed WB assay or subjected to Transwell/migration assay. *P< 0.05, relative to NaCl treatment of the same cell type as controls, ^#^*P*< 0.05, relative to that of Vector with the same treatment as controls. (**e** and **f**) Cells were treated with 100 ng/ml Wnt3a in the absence or presence of PEDF for 48 h, and then performed WB assay or subjected to Transwell/migration assay. Bars correspond to mean±standard deviation (S.D.), *P<0.05

We have previously reported that PEDF serves as a novel inhibitor of the canonical Wnt pathway, binds to Wnt co-receptor low-density lipoprotein receptor-related protein 6 (LRP6) and blocks the signaling by antagonizing Wnt ligands in retinal pigment epithelial cells.^19^ A higher level of nuclear *β*-catenin was found in high-metastatic S18 and 5–8 F cells compared with low-metastatic CNE-2 and SUNE-1 cells (Supplementary Figures 1A and B). Consequently, we validate whether Wnt/*β*-catenin signaling pathway is responsible for the inhibitory effect of PEDF in NPC. To further confirm a direct modulation of Wnt/*β*-catenin by PEDF, we evaluate the effect of PEDF on the nuclear translocation of *β*-catenin. PEDF could decrease the elevated nuclear *β*-catenin level in S18 and 5–8 F cells (Figure 5a). The subcellular fractionation results also confirmed that PEDF could decrease the nuclear *β*-catenin levels (Figure 5b). Strikingly, the inhibited EMT and migration in NPC cells caused by overexpressing PEDF were restored by LiCl, which was used to activate *β*-catenin (Figures 5c and d). Next, we used Wnt3a, the canonical Wnt/*β*-catenin pathway ligand, to activate the Wnt/*β*-catenin pathway and found that Wnt3a could increase the total Wnt co-receptor LRP6 level and decrease the expression of E-cadherin thus promote the cell migration, while these functions could be reversed by PEDF. Furthermore, PEDF-inhibited Wnt3a activation was abolished by Wnt/*β*-catenin signaling intracellular agonist LiCl (Figures 5e and f), which suggests that PEDF regulates Wnt signaling at the receptor level. These results showed that inhibition of Wnt/*β*-catenin signaling pathway is required for PEDF-inhibited EMT in NPC.

### PEDF is a direct target of miR-320c in NPC

**Figure 6:**
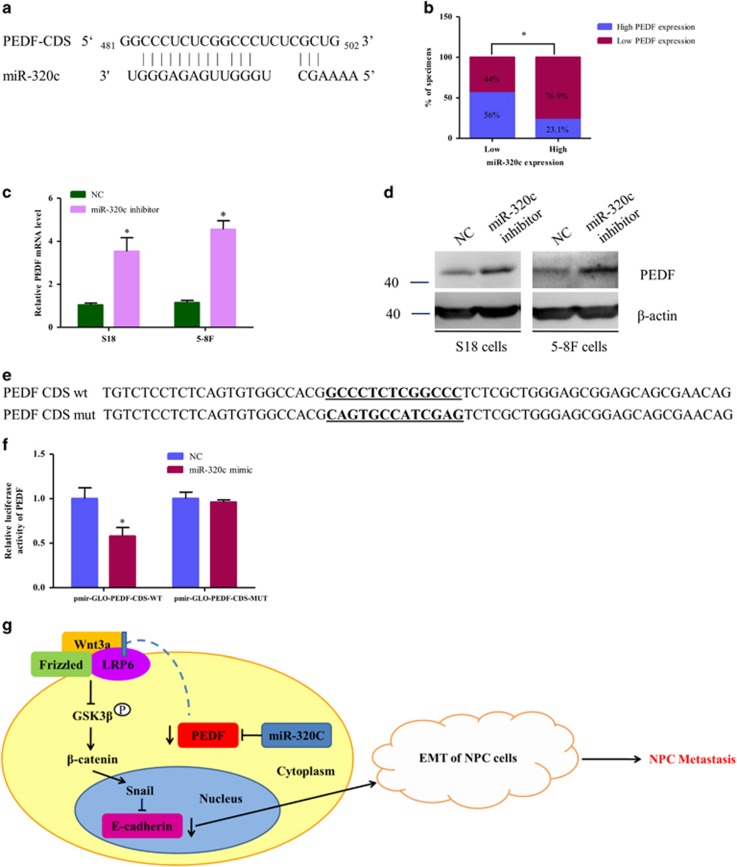
PEDF is a direct target of miR-320c. (**a**) Schematic miR-320c putative target sites in the CDS of PEDF. (**b**) Percentage of specimens showing low or high miR-320c expression with the expression levels of PEDF. (**c** and **d**) Cells were transfected with miR-320c inhibitors for 48 h, and then performed WB or qRT-PCR analysis. (**e**) The sequence of PEDF-mut. (**f**) Indicated plasmids were co-transfected with NC or miR-320c mimics, respectively, for 48 h and then performed luciferase reporter assay, pmir-GLO was transfected as the internal control. (**g**) The schematic overview of the potential mechanism involved in PEDF-mediated EMT in NPC. Bars correspond to mean±standard deviation (S.D.), *P<0.05

As PEDF has an important role in NPC progression, the reason for the diminished PEDF observed in NPC is unclear. We have previously found miR-320c was increased in the plasma of NPC patients,^21^ and miRNA target prediction algorithms (RNAhybrid) also identified that there is a potential binding sites of miR-320c in PEDF coding sequence (CDS) region^22^ (Figure 6a). We asked whether miRNA could be responsible for PEDF regulation. The expression level of PEDF and miR-320c was evaluated using qRT-PCR in 11 NET tissues and 27 NPC samples. As shown in Figure 6b, 56% (14 cases) of samples with low miR-320c expression (25 cases) exhibited high levels of PEDF, whereas 76.9% (10 cases) of samples with high miR-320c expression (13 cases) showed low expression of PEDF (*P*<0.05), which indicated that the expression levels of miR-320c were inversely related to the expression levels of PEDF in nasopharynx tissues. Furthermore, western blot and qRT-PCR showed that inhibition of miR-320c increased the endogenous PEDF expression in the NPC cell lines S18 and 5–8 F (Figures 6c and d). Furthermore, reporter assays showed that the activity of luciferase linked with the CDS of PEDF was repressed in miR-320c mimic–transfected 293A cells, compared with those in control cells. Of note, mutations brought into the CDS of PEDF abolished miR-320c suppressive effects (Figures 6e and f). Collectively, these data suggest that miR-320c directly suppresses PEDF expression by targeting CDS of PEDF.

## Discussion

NPC has the highest rate of invasive and metastasis among head and neck malignant tumor, and distant metastasis is the leading cause of treatment failure.^23^ Therefore, clarifying the underlying mechanism of nasopharyngeal carcinoma metastasis and developing effective targeting drugs became the core issue for the treatment of NPC. The present study provides clinical and experimental evidences to support the tumor-suppressor role of PEDF in NPC. Our results revealed that the deficiency of PEDF is involved in the occurrence of metastasis in NPC patients and confirmed that knockdown of PEDF induces EMT of NPC cells and promotes invasiveness, whereas restoration of PEDF reverses EMT phenotype of NPC. We further demonstrated that PEDF suppress EMT and metastasis depended on its inhibitory effect on Wnt/*β*-catenin pathway. Finally, we verified that the increased miR-320c level in NPC direct downregulates expression of PEDF. Previously, we have reported that PEDF could combine with radiotherapy to enhance the antitumor effects on NPC.^24^ Based on these findings, we believe that PEDF could be an appealing candidate for prevention and treatment of NPC progression.

It has been reported that the distinct oncogenes were activated in the EMT induction of NPC. Epstein–Barr virus induces the initiation, metastasis and recurrence of NPC through its encoded protein LMP1 and LMP2A.^5, 6^ Bmi-1 has an important role in the pathogenesis of NPC by inducing EMT via the PI3K/Akt pathway.^25^ However, tumor-suppressor genes involved in the EMT and metastasis of NPC have rarely been identified. To our knowledge, we demonstrate for the first time that PEDF was downregulated in human NPC tissues compared with NET tissues (Figure 1a). Only 7.3% (16/218) of NPC tumor tissues had PEDF weak expression (Figure 1b). These results suggest that the loss of PEDF might occur in the early stage of NPC. We also observed that NPC patients of negative PEDF expression had advanced pathological tumor stage and clinical stage (Table 1). Furthermore, the loss of PEDF could induce EMT occurrence (Figures 2, 3, 4). Taken together, our data provide a biological basis for the feasible role of PEDF as a tumor-suppressor gene in NPC.

In this study, our results identify that PEDF participates in NPC cells’ EMT *in vitro* (Figures 2 and 3). Importantly, this effect was reproduced *in vivo* (Figure 4 and Supplementary Figure 2). To our knowledge, this is the first study demonstrating an essential role for PEDF in inhibition of EMT. Previous studies showed PEDF is a useful marker for monkey RPE polarization and increased in polarized human RPE cells compared with non-polarized RPE cells while losing apical-basal polarity is the important step during EMT.^18, 26, 27^ Orgaz *et al.*^28^ showed that PEDF expression was correlated with E-cadherin and N-cadherin in some pairing melanoma cells but not SBcl2 cells. Downregulation of PEDF is associated with increased EMT in bladder and breast cancer tiusses.^29, 30^ PEDF overexpression in MD-231 breast cancer cells decreased fibronectin and migration but did not change the EMT phenotype in our recent study.^31^ This cell type-specific function has been observed with other proteins, such as Bmi-1 and ILEI.^25, 32^ Thus, PEDF-participated EMT may be existed in a cell type-dependent manner.

Cellular signaling pathways, including the Wnt/*β*-catenin,^33, 34, 35^ NF-*κ*B^36, 37^ and PI3K/Akt^6, 38^ pathways have been found to be aberrantly activated and have vital roles in the development and progression of NPC. In this study, we observed increased phosphorylation of GSK3*β* and decreased snail in PEDF-overexpressed NPC cells (Supplementary Figure 3A). Furthermore, the restored epithelial marker and decreased cellular motility triggered by PEDF overexpression were eliminated by GSK3*β* inhibitor CHIR99021 (Supplementary Figures 3B and C). Phosphorylation of GSK3*β* could be regulated by PI3K/Akt, MAPK and Wnt/*β*-catenin.^39^ PEDF decreased *β*-catenin and nuclear translocation in high-metastatic S18 and 5–8 F cells (Figures 5a and b). However, no phosphorylation changes of Akt and MAPK were observed in the PEDF-treated S18 and 5–8 F cells (Supplementary Figure 3A). Thus, the GSK3*β*-mediated Wnt/*β*-catenin pathway rather than Akt pathway seems to have a critical role in PEDF-inhibited EMT.

It is believed that the diminished PEDF expression in various tumor types may in part account for increased malignant characteristics during tumor progression.^40^ Possible regulatory mechanisms underlying the changes in PEDF expression during transforming and malignant progression of cancer are rarely studied, which include transcriptional regulation, hypoxia-mediated regulation and posttranslational modifications.^41^ Our findings suggested that PEDF is suppressed by miRNAs in NPC, indicative of a novel mechanism of PEDF regulation (Figure 6). To assess the causative role of miR-320c in the loss of PEDF, miR-320c inhibitors were infected into high-metastatic S18 and 5–8 F cells. The results showed both of *β*-catenin and EMT are inhibited by miR-320c inhibitors (Supplementary Figures 4A and B). Furthermore, pathological tissue analyses demonstrated that miR-320 was increased in many types of cancer, including breast cancer, retinoblastoma and neuroblastoma, while PEDF is less in this kind of cancer types,^42, 43, 44^ which was similar to our findings. Moreover, previous studies also revealed that miR-320 with the pro-angiogenic function is decreased in type 2 diabetes.^45, 46^ These results indicate that PEDF may be regulated by miR-320c in different types of diseases. Interestingly, in this study, PEDF was regulated by miR-320c at its CDS site rather than 3′-UTR (Supplementary Figures 5A-C). The detail points for the regulation of miR-320c on PEDF in CDS site needs to be explored in the future.

PEDF has been found to have prognostic value in various types of malignancies, including colorectal cancer, pancreatic cancer, lung cancer and breast cancer.^14, 15, 16, 17^ In this study, we observed PEDF have prognostic value in 42 months survival of NPC patients. However, we failed to identify a significant relation between PEDF and patient outcomes in NPC patients’ 5 years survival (Supplementary Table 4). This may be due to only 7.3% (16/218) PEDF-positive expression (Figure 1b) and the 5 years survival of NPC patients is very high (158/218, 72.4%). We believe it is important to perform another clinical study with a larger cohort of PEDF-positive specimens and longer follow-up time to further clarify the correlation between PEDF expression and patient outcomes. But, even so, our data demonstrated that the expression pattern of PEDF was remarkably different between NET and NPC, which indicates that PEDF could be as a clinical adjuvant diagnosis marker of NPC.

In summary, PEDF is markedly decreased in NPC tissues and correlated with clinicopathological features. Deficiency of PEDF in nasopharyngeal carcinoma cells triggers the EMT and metastasis *in vitro* and *in vivo* via activation of Wnt/*β*-catenin pathway. The downregulation of PEDF was regulated by miR-320c directly in NPC cells. These findings have raised a new mechanism for the EMT and metastasis of NPC, proposed a new function of PEDF and suggested PEDF serving as a potential diagnosis, treatment and prognosis candidate for nasopharyngeal carcinoma.

## Materials and Methods

### Clinical samples

The tissue samples of nasopharyngeal cancer (NPC; *n*=218) or nasopharynx epithelial tissues (NET; *n*=12) used in Figures 1a and b and Table 1 were provided by the Department of Molecular Diagnostics (Sun Yat-sen University Cancer Center, Guangzhou, China). For Figures 1c–e and Figure 6b, paraffin-embedded tissues from a total of 12 normal controls and 20 NPC tissues, and fresh frozen samples of NET (*n*=11) or NPC (*n*=27) were provided by the cancer center (Affiliated Hospital of Guangdong Medical College, Zhanjiang, China). None of the patients had received any chemotherapy or radiotherapy before their operation. Data of clinicopathological parameters were obtained from patients' clinical records and pathological reports. This study conforms to the principles outlined in the Declaration of Helsinki, approved by the Medical Ethics Committee of Sun Yat-Sen University and Guangdong Medical College. Written informed consents were obtained from the donors.

### Immunohistochemical staining

Paraffin-embedded tissues were sectioned at 5 *μ*m and immunohistochemistry assays were performed. Briefly, the sections were hydrated, blocked and incubated with a series of antibodies overnight at 4 °C. The Dako real EnVision kit (Dako, Santa Clara, CA, USA) was used to detect the primary antibodies followed by 3, 3-diaminobenzidine substrate visualization and counterstaining with hematoxylin. The results were evaluated by assessing staining intensity according to the method described by Remmele and Stegner.^47^ The average value from two referees was used as the final score.

### Cell culture

The NPC cell lines CNE-2 and its subclones S18 were provided by Professor Qian Chao-nan.^38^ The SUNE-1 and its subclones 5–8 F were the kind gifts of Professor Zeng Mu-sheng at Sun Yat-sen University Cancer Center.^10^ All cell lines were cultured for less than 50 passages. The NPC cell lines were maintained in RPIM 1640 medium (Hyclone, Beijing, China) supplemented with 10% fetal bovine serum (Gibco, Montevideo, Uruguay). All the cell lines were incubated at 37 °C in a 5% CO_2_ incubator. For 3D cell culture, 1 × 10^4^ cells were seeded on Matrigel-coated 24-well plates and the complete medium containing 2% Matrigel was refreshed every day. The cells forming spheres were photographed at 2-day intervals for 8 days.

### RNA extraction and qRT-PCR

Small RNA was extracted from samples using RNAiso Kit for Small RNA (TaKaRa, Dalian, China) and subsequently reverse transcribed into cDNA by One Step PrimeScript miRNA cDNA Synthesis Kit (TaKaRa). Meanwhile, total RNA from samples was extracted using Ultrapure RNA Kit (KangWei, Beijing, China) and transcribed into cDNA using PrimeScript RT reagent Kit (TaKaRa). The sequences of PCR primers were shown in Supplementary Table 1. The quantitative PCR was performed using SYBR Premix Ex Taq (TaKaRa) and a LightCycler 2.0 Real Time PCR system (Roche Diagnostics, Rotkreuz, Switzerland). U6 and *β*-actin were used for normalizing the expression of miRNA and mRNA, respectively. The experiments were performed in triplicate.

### Lentiviral transduction studies

Cell lines stably expressing PEDF were established using a ViraPower Lentiviral Packaging Kit (Invitrogen, Carlsbad, CA, USA) according to the manufacturer’s instructions. Briefly, oligos containing the sequences of PEDF were cloned into a lentiviral vector. The lentiviral particles were prepared by transient co-transfection of the resulting vector and ViraPower Lentiviral Packaging Mix into 293FT cells using Lipofectamine 2000 (Invitrogen). Infectious lentiviruses were harvested 72 h after transfection, centrifuged to remove cell debris, filtered through 0.45 *μ*m filter (Millipore, Bedford, MA, USA) and concentrated by centrifugal filter device (Millipore). Lentiviruses expressing either PEDF short hairpin RNA (shPEDF) or a Scrambled nontarget shRNA were established by Genechem company. The target for human PEDF shRNA was 5′-GGATCGTCTTTGAGAAGAA-3′. The NPC cells were transduced with lentiviruses. After 7 days, 1 *μ*g/ml puromycin (Sigma-Aldrich, St Louis, MO, USA) was added to the culture and lasted for 10 days. Western blot and qRT-PCR were performed to analyze the PEDF expression.

### Western blot

The cells were washed with cold PBS and collected using the RIPA lysis buffer (Santa Cruz, St, Dallas, TX, USA). Nuclear extracts were obtained using the NE-PER Nuclear and cytoplasmic extraction reagents (Thermo, Rockford, AL, USA) according to the manufacturer's instructions. The concentration of total protein in the lysate was measured using the Bio-Rad protein assay kit (Bio-Rad, Hercules, CA, USA). An equal amount of protein in each sample was loaded onto 12% SDS-PAGE gels and transferred to PVDF membranes (Roche Diagnostics). The membrane was probed with the first antibody listed in Supplementary Table 2 at 4 °C overnight and then with the peroxidase-conjugated secondary antibody, detected with ECL kit (APPLYGEN, Beijing, China). The same membrane was stripped and incubated with an anti-*β*-actin antibody for normalization.

### Migration and invasion assays

A total of 2 × 10^4^ (for S18 and 5–8 F) or 4 × 10^4^ (for CNE-2 and SUNE-1) cells in 200 *μ*l serum-free RPIM 1640 were seeded on cell culture inserts with 8 *μ*m microporous filters (Corning, Corning, NY, USA) coated with (invasion) or without (migration) Matrigel (BD Biosciences, Franklin Lakes, NJ, USA), and 800 *μ*l of RPIM 1640 containing 10% FBS was added to the lower chamber. After being incubated for indicated times, the cells in the upper filters (inside the inserts) were removed, and the migrated or invaded cells in the lower filters (outside the inserts) were fixed in ethanol for 20 min, then stained with crystal violet for 10 min and counted under a microscope. The number of migrated or invaded cells in five random optical fields (× 100 magnification) of each filter from triplicate inserts was averaged. Experiments were performed in triplicate.

### Immunofluorescence analysis

The cells were seeded on coverslips and incubated for 24 h, washed with cold PBS three times and fixed with ice-cold paraformaldehyde for 10 min at −20 °C. Next, the cells were permeabilized for 10 min with 0.01% Triton X-100 in PBS. Then the cells were blocked for 30 min in normal goat serum and incubated with the anti-*β*-catenin antibody in antibody diluents (Dako) overnight at 4 °C. After washing with PBS three times, the slides were incubated for 1 h in the dark at 37 °C with the Rhodamine-conjugated secondary antibody (Sigma-Aldrich). After three further washes, the slides were stained with 4′,6-diamidino- 2-phenylindole (DAPI, Sigma-Aldrich) for 10 min to counterstain the nucleus, and examined using the Axio Imager Z1 microscope (ZEISS, Oberkochen, Baden-Wuerttemberg, Germany). In negative control staining, the primary antibodies were omitted.

### Animals and liver metastasis assay

BALB/c-nu male mice (16–18 g), at age 5–6 weeks, were purchased from the Laboratory Animal Center of Guangdong Province (Guangzhou, China) and fed in a specific pathogen-free environment. The mice were randomly grouped into two groups (five mice/group). The NPC cells (2 × 10^5^ in 30 *μ*l of 33% Matrigel) were injected into the spleens of laparotomized nude mice using insulin syringes (BD Biosciences) as previously reported.^38^ After 28 days, the experiment was terminated. The liver and spleen of each mouse were weighed and the metastatic nodules in each liver were counted. The sections from paraffin-embedded samples were performed H&E staining for histological evidence of the tumor phenotype. The care, use and treatment of all animals were in strict agreement with the institutionally approved protocol according to the USPHS Guide for the care and use of laboratory animals, as well as the guidelines outlined in Care and Use of Laboratory Animals by the Sun Yat-sen University in this study.

### Luciferase reporter assay

A total 1 × 10^4^ cells were seeded in triplicate in 96-well plates. Indicated plasmids plus NC or miR-320c mimics were transfected into the 293 A cells using Lipofectamine 2000 (Invitrogen). Forty-eight hours post transfection, Dual-Glo Luciferase Assay System (Promega, Madison, SD, USA) was performed according to the manufacturers’ instruction. Renilla luciferase assay was used to normalize the corresponding firefly luciferase activity.

### Statistical analysis

Differences among variables were assessed by two-tailed Student’s unpaired *t*-tests. The categorical data were analyzed by the Chi-square method. Data were presented as the mean±standard deviation (S.D.). *P*<0.05 was considered statistically significant. The statistical analyses were performed using SPSS 18.0 (Chicago, IL, USA) or GraphPad Prism 5.0.

## Figures and Tables

**Table 1:** Correlation between PEDF and clinicopathologic characteristics of patients with NPC

		**PEDF expression level**		
**Characteristic**	**Number**	**Positive**	**Negative**	***P*****-value**
*T stage*				***0.018***
T1–2	99	12 (12.1%)	87 (87.9%)	
T3–4	119	4 (3.3%)	115 (96.7%)	
				
*N stage*				0.292
N0–1	132	12 (9.1%)	120 (90.9%)	
N2–3	86	4 (4.7%)	82 (95.3%)	
				
*Clinical staging*				***0.045***
I–II	68	9 (13.2%)	59 (86.8%)	
III–IV	150	7 (4.7%)	143 (95.3%)	

*P*<0.05 were presented in bold and italic.
