# Correction: Deficiency of pigment epithelium-derived factor in nasopharyngeal carcinoma cells triggers the epithelial–mesenchymal transition and metastasis

**DOI:** 10.1038/s41419-018-0829-x

**Published:** 2018-07-16

**Authors:** Ting Zhang, Ping Yin, Zichen Zhang, Banglao Xu, Di Che, Zhiyu Dai, Chang Dong, Ping Jiang, Honghai Hong, Zhonghan Yang, Ti Zhou, Jianyong Shao, Zumin Xu, Xia Yang, Guoquan Gao

**Affiliations:** 1 Program of Molecular Medicine, Affiliated Guangzhou Women and Children’s Hospital, Zhongshan School of Medicine, Sun Yat-sen University, Guangzhou, 510080 China; 2 Department of Biochemistry, Zhongshan School of Medicine, Sun Yat-sen University, Guangzhou, 510080 China; 3 Department of Laboratory Medicine, Guangzhou First People’s Hospital, Guangzhou Medical University, Guangzhou, 510180 China; 4 Department of Molecular Diagnostics, Sun Yat-sen University Cancer Center, Guangzhou, 510160 China; 5 Department of Clinical Laboratory, Third Affiliated Hospital of Guangzhou Medical University, Guangzhou, 510150 China; 6 Cancer Center, Affiliated Hospital of Guangdong Medical College, Zhanjiang, 524001 China; 7 Guangdong Engineering & Technology Research Center for Gene Manipulation and Biomacromolecular Products (Sun Yat-sen University), Guangzhou, 510080 China; 8 China Key Laboratory of Tropical Disease Control (Sun Yat-sen University), Ministry of Education, Guangzhou, 510080 China

Correction to: *Cell Death* & *Disease.* 10.1038/cddis.2017.114; published online 1 June 2017.

The PDF and HTML versions of the article have been updated to include the Creative Commons Attribution 4.0 International License information.

