# Supplementary material for: Deficiency of pigment epithelium-derived factor in nasopharyngeal carcinoma cells triggers the epithelial–mesenchymal transition and metastasis
Source: Cell Death Dis. 2017 Jun 1;8(6):e2838–. doi: 10.1038/cddis.2017.114 (PMC5520876; doi:10.1038/cddis.2017.114)
Supplement: Supplementary Information [file cddis2017114x1.pdf]

Figure S1

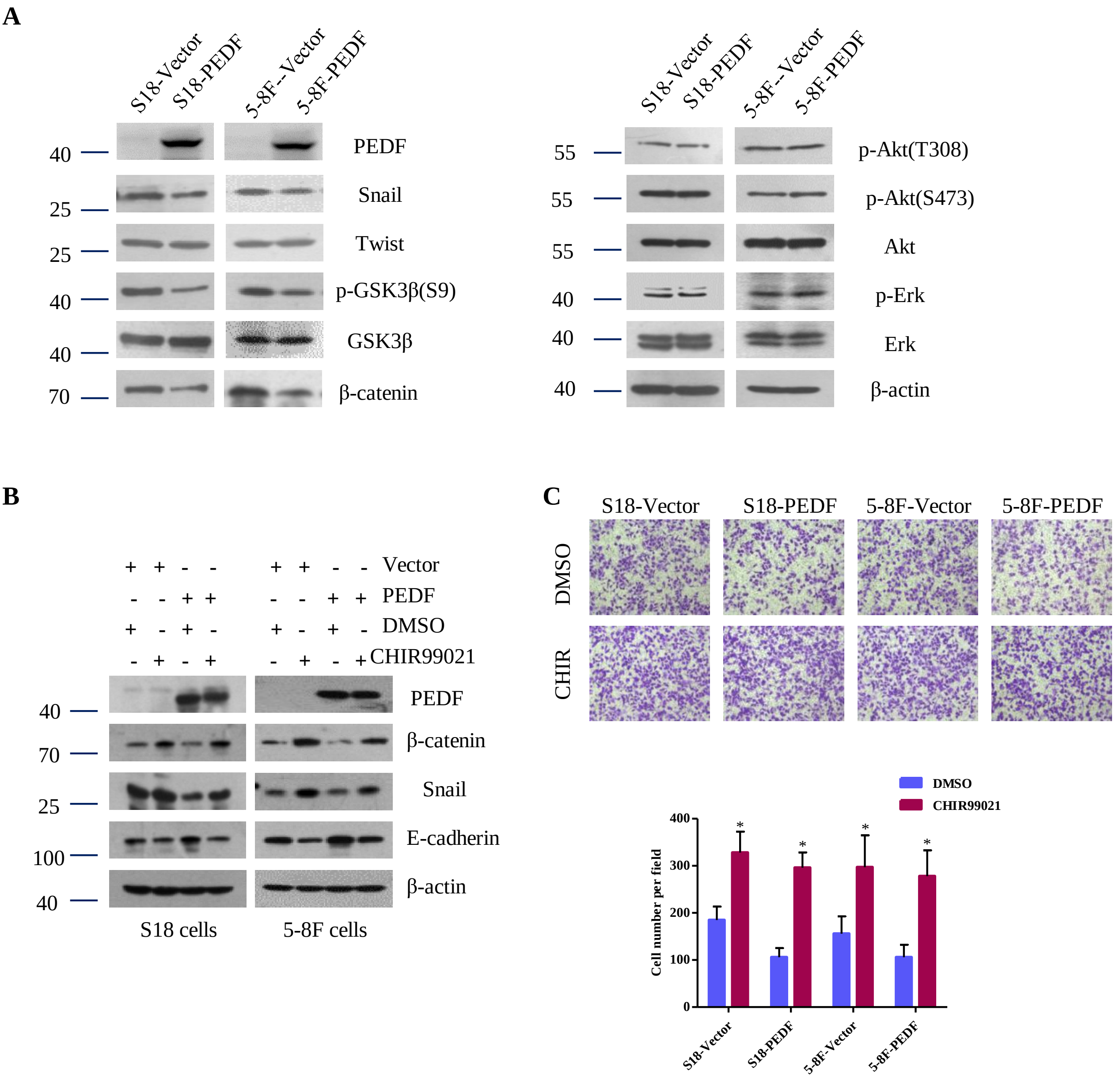
A
S18-Vector
S18-PEDF
5-8F--Vector
5-8F-PEDF
PEDF
Snail
Twist
p-GSK3β(S9)
GSK3β
β-catenin
40
25
25
40
40
70
p-Akt(T308)
p-Akt(S473)
Akt
p-Erk
Erk
β-actin
55
55
55
40
40
40
B
Vector
PEDF
DMSO
CHIR99021
PEDF
β-catenin
Snail
E-cadherin
β-actin
40
70
25
100
40
S18 cells
5-8F cells
C
S18-Vector
S18-PEDF
5-8F-Vector
5-8F-PEDF
DMSO
CHIR
DMSO
CHIR99021
Cell number per field
0
100
200
300
400
*
S18-Vector
S18-PEDF
5-8F-Vector
5-8F-PEDF

Figure S2

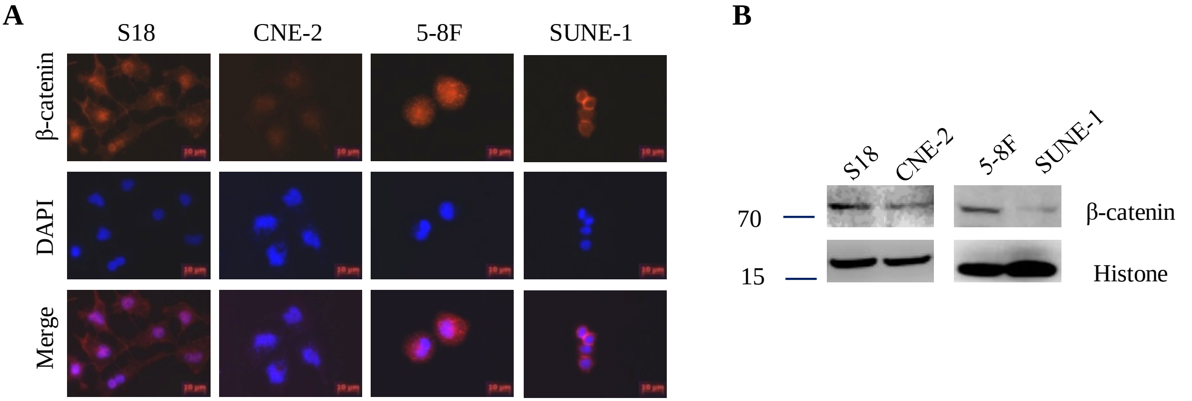
A
S18
CNE-2
5-8F
SUNE-1
β-catenin
DAPI
Merge
B
S18
CNE-2
5-8F
SUNE-1
70
15
β-catenin
Histone

Figure S3

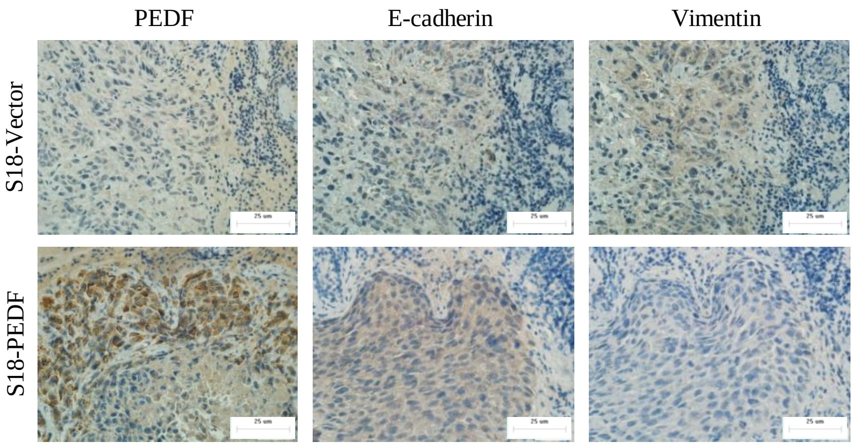

Figure S4

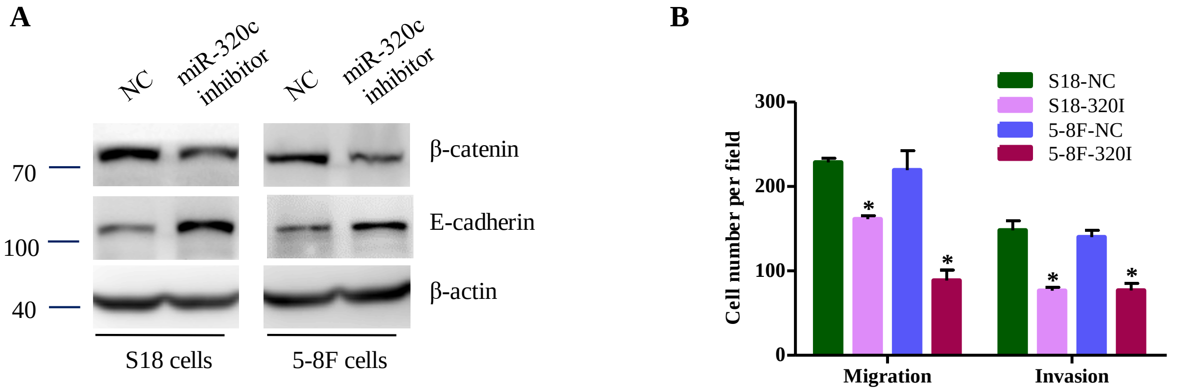
A
NC
miR-320c inhibitor
NC
miR-320c inhibitor
70
100
40
β-catenin
E-cadherin
β-actin
S18 cells
5-8F cells
B
S18-NC
S18-320I
5-8F-NC
5-8F-320I
Cell number per field
300
200
100
0
*
*
*
*
Migration
Invasion

Figure S5

A

PEDF-3’UTR 5‘ $_{91}$ UGCAUACAAUAAAAGAGCUUUAU$_{114}$ 3’
| | | | | |
miR-320c 3‘ UGGGAGAGUUGGGUCGAAAA 5’

B

PEDF 3’UTR wt TGTCTCCTCTCAGTGTGGCCACG**AGCTTT**TCTCGCTGGGAGCGGAGCAGCGAACAG

PEDF 3’UTR mut TGTCTCCTCTCAGTGTGGCCACG**CATGTT**TCTCGCTGGGAGCGGAGCAGCGAACAG

C

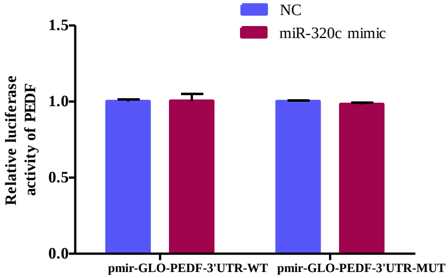

Supplementary Table 1. The sequences of PCR primers

PEDF forward	5′-CAGAAGAACCTCAAGAGTGCC-3′
PEDF reverse	5′-CTTCATCCAAGTAGAAATCC-3′
β-actin forward	5′-GCACTCTTCCAGCCTTCCTT-3′
β-actin reverse	5′-GTTGG CGTACAGGTCTTTGC-3′
miR-320c forward	5′- AAAAGCTGGGTTGAGAGGGT -3′
U6 forward	5′- TGCGGGTGCTCGCTTCGGCAGC -3′
E-cadherin forward	5′-CCCACCACGTACAAGGGTC-3′
E-cadherin reverse	5′-CTGGGGTATTGGGGGCATC-3′
α-catenin forward	5′-CCTGCTGGCCTACCTGCAACG-3′
α-catenin reverse	5′-CATGGCGCTGTCCACCCCAGA-3′
N-cadherin forward	5′-TCCACGCCGAGCCCCAGTAT-3′
N-cadherin reverse	5′-TCAGCCGCTTTAAGGCCCTCAT-3′
Vimentin forward	5′-GGCCGCCTGCAGGATGAGATT-3′
Vimentin reverse	5′-AGAAATCCTGCTCTCCTCGCCTT-3′

Supplementary Table 2. The antibodies used for western blot and IHC staining

Name of antibody	Cat. No	Company	Dilution(WB/IHC)
PEDF	MAB1059	Merck Millipore	1:1000/1:100
E-cadherin	610181	BD	1:2000/1:1000
α-catenin	610193	BD	1:2000
N-cadherin	610921	BD	1:2000
Vimentin	550513	BD	1:3000/1:2000
GSK-3β	610202	BD	1:1000
p-GSK-3β（Ser9）	#9323	CST	1:1000
Akt	#4691S	CST	1:1000
p-Akt (Ser473)	#4060S	CST	1:1000
β-catenin	#9582	CST	1:1000
Snail	#3879	CST	1:1000

Supplementary Table 3. Clinical characteristics of 218 NPC patients

Characteristics	No. (%)
**Age, years**	
Median	46
Range	18-76
**Gender**	
male	163(74.8)
female	55(25.2)
**WHO histological type**	
Type I	5(2.3)
Type II	20(9.2)
Type III	193(88.5)
**T stage**	
T1	13(6.0)
T2	86(39.4)
T3	74(34.0)
T4	45(20.6)
**N stage**	
N0	57(26.1)
N1	75(34.4)
N2	67(30.7)
N3	19(8.8)
**M stage**	
M0	218(100.0)
M1	0(0.0)
**Clinical staging**	
I	4(1.8)
II	64(29.4)
III	88(40.4)
IV	62(28.4)
**Treatment outcome**	
**Death**	
Yes	60(27.5)
No	158(72.5)
**Progression**	
Yes	23(10.6)
No	195(89.4)

**Distant metastasis**	
Yes	11(5.0)
No	207(95.0)
**Loco-regional relapse**	
Yes	13(6.0)
No	205(94.0)

WHO histological type, Type I: well-differentiated keratinizing squamous cell carcinoma; Type II: non-keratinizing squamous cell carcinoma; Type III: undifferentiated carcinoma.

Supplementary Table 4. Association of PEDF expression and clinicopathological characteristics of patients with NPC

Characteristic	Number	PEDF expression level		*P* value
		Positive	Negative	(Chi-square test)
**Gender**				1.0
Male	163	12	151	
Female	55	4	51	
**Age**				0.198
≤46	118	6	112	
>46	100	10	90	
**T stage**				***0.018***
T1-2	99	12	87	
T3-4	119	4	115	
**N stage**				0.292
N0-1	132	12	120	
N2-3	86	4	82	
**M stage**				NA
M0	218	16	202	
M1	0	0	0	
**Clinical staging**				***0.045***
I-II	68	9	59	
III-IV	150	7	143	
**WHO histological type**				0.226
Type I	193	16	177	
Type II-III	25	0	25	
**42months survival**				***0.047***
Yes	44	0	44	
No	174	16	158	
**5 years survival**				1.0
Yes	60	4	56	
No	158	12	146	
**Disease progression**				0.229
Yes	23	0	23	
No	195	16	178	
**Distant metastasis**				1.0
Yes	11	0	11	
No	207	16	191	

**Loco-regional relapse**				0.605
Yes	13	0	13	
No	205	16	189	

Supplementary Table and Figure Legends

Supplementary Figure 1. GSK3β mediates PEDF-inhibited EMT in NPC cells.

(A) Cell extracts were analyzed by immunoblotting with antibodies against the indicated proteins. (B, C) Cells were treated with GSK3β inhibitor CHIR99021 (5 μM) for 24 h, and then performed WB assay or subjected to Transwell/migration assay. Experiments in B, C were repeated at least 3 times. Bars correspond to mean ± standard deviation (SD), *P<0.05.

Supplementary Figure 2. β-catenin is abnormally activated in high metastatic NPC cell lines.

(A) Subcellular β-catenin localization in indicated cells was assessed by immunofluorescence staining. (B) Nuclear fractions of indicated cells were analyzed by WB analysis.

Supplementary Figure 3. PEDF inhibits the EMT of NPC cells *in vivo*. Immunohistochemical staining of ectopic E-cadherin and Vimentin expression in harvested mice spleen samples.

Supplementary Figure 4. Down-regulated of endogenous miR-320c in NPC cells inhibits Wnt/β-catenin signaling pathway and EMT.

(A, B) Cells were transfected with miR-320c inhibitors for 48 h, and then performed WB assay or subjected to Transwell/migration assay. Experiments in A, B were repeated at least 3 times. Bars correspond to mean ± standard deviation (SD), *P<0.05.

Supplementary Figure 5. miR-320c could not bind to PEDF 3'UTR.

(A) Schematic miR-320c putative target sites in the 3'UTR of PEDF. (B) The sequence of PEDF-mut. (C) Indicated plasmids were co-transfected with NC or miR-320c mimics respectively for 48 h and then performed luciferase reporter assay, pmir-GLO was transfected as the internal control. Experiments in C were repeated at least 3 times. Bars correspond to mean ± standard deviation (SD), *P<0.05.

Supplementary Table 1. The sequences of PCR primers.

Supplementary Table 2. The antibodies used for western blot and IHC staining.

Supplementary Table 3. Clinical characteristics of 218 NPC patients.

Supplementary Table 4. Association of PEDF expression and clinicopathological characteristics of patients with NPC.
